# Improving governance of IVF and oncofertility practice in the USA: a call to action

**DOI:** 10.1093/hropen/hoag033

**Published:** 2026-04-16

**Authors:** Mahmoud Salama, Teresa K Woodruff

**Affiliations:** The Oncofertility Consortium, Department of Obstetrics, Gynecology and Reproductive Biology, Michigan State University, East Lansing, MI, USA; The Oncofertility Consortium, Department of Obstetrics, Gynecology and Reproductive Biology, Michigan State University, East Lansing, MI, USA

Dear Editor,

As reported recently in *Human Reproduction Open* by Gleicher *et al*., there has been a steady decline in IVF cycle efficiency with live birth rate dropping from 29.1% to 22.2% between 2012 and 2021 in the USA despite an increase in overall IVF cycle numbers by 234.7% (Gleicher *et al*., 2026). These findings have garnered growing attention from infertility experts, as described by Gleicher *et al*., and are of deep concern to those practitioners in the oncofertility community.

Oncofertility is an interdisciplinary field at the intersection of oncology and reproductive medicine offering several assisted reproduction strategies including cryopreservation of gametes and gonadal tissue in order to preserve fertility in young patients with cancer before the initiation of gonadotoxic chemotherapy and radiotherapy (Woodruff *et al*., 2021). Therefore, any decline in IVF efficiency may result in permanent fertility loss. Indeed, unique challenges exist for oncofertility patients making them more vulnerable to inefficiencies in assisted reproduction services. The primary challenge is the very limited timeframe between cancer diagnosis and treatment. This time-sensitive window, which may be just a few weeks or even days, should accommodate proper patient counselling and decision-making on fertility preservation, and in many cases only one chance for participation in a cryopreservation intervention. This sophisticated clinical workflow for oncofertility patients requires a best practice approach, including immediate referral and high-level coordination between a multidisciplinary team of oncologists and reproductive endocrinology and infertility (REI) specialists to assess the risk of gonadotoxicity and design the most suitable fertility preservation strategy for the patient. Other unique challenges for oncofertility patients include a shortage of oncofertility services, lack of awareness among providers and patients, cultural and religious beliefs regarding survivorship, lack of insurance coverage, and lack of funding to support oncofertility programs resulting in high out-of-pocket costs for patients (Woodruff and Salama, 2025).

Declining IVF cycle efficiency in the USA despite an increase in overall IVF cycle numbers raises governance concerns for infertility and oncofertility patients as most fertility preservation services, such as gametes cryopreservation, are offered within the same commercial settings at many IVF centers. Better governance of IVF practices will benefit both infertility and oncofertility patients and could be achieved through re-evaluating IVF practices and clinical sites, enhancing transparency and accurate reporting to the Centers for Disease Control and Prevention (CDC), and strengthening the evidence thresholds for add-on services that may require additional technical skills and incur extra costs.

We thank Gleicher *et al*. (2026) for highlighting this critical topic and we join their call for re-evaluating the current IVF practices to ensure that both infertility and oncofertility patients are not negatively impacted by system-level inefficiencies.

## Editor’s note

The authors of Gleicher *et al*. (2026), the original article to which this Letter to the Editor refers, were offered the opportunity to publish a Reply Letter to the Editor in response. The authors voiced their appreciation of the Letter and, after further consideration, agreed there was no need to provide a formal response.
